# 1819. Characterizing Treatment Duration of Viridans Group Streptococci Bacteremia in Neutropenic Cancer Patients: Is 14 Days Necessary?

**DOI:** 10.1093/ofid/ofac492.1449

**Published:** 2022-12-15

**Authors:** Yaling Vu, Jenny Wong, Catherine Liu, Frank P Tverdek

**Affiliations:** University of Washington, Seattle, Washington; University Of Washington, Seattle, Washington; Fred Hutchinson Cancer Research Center, Seattle, Washington; Seattle Cancer Care Alliance, Seattle, Washington

## Abstract

**Background:**

Neutropenic oncology patients often have mucosal barrier injury as a consequence of chemotherapy and are at risk of Viridans Group Streptococci (VGS) bacteremia. VGS is a commonly identified pathogen among oncology patients with neutropenic fever, however, limited data exists as to appropriate treatment duration.

**Methods:**

The purpose of this study is to characterize the treatment duration and clinical course among neutropenic oncology patients with VGS bacteremia. A retrospective chart review of patients with VGS bacteremia from June 1, 2020 through January 31, 2022 at the University of Washington Medical Center (UWMC) and Seattle Cancer Care Alliance (SCCA). Eligible patients include adults ≥ 18 years with any cancer diagnosis, absolute neutrophil count ≤ 500 at time of blood culture collection, ≥ 1 positive blood culture for VGS, and treated with antibiotics for ≥ 48 hours. Polymicrobial infections were excluded.

**Results:**

Among 86 patients identified with VGS bacteremia, 57 patients were included. Baseline characteristics are depicted in TABLE 1. Hematologic malignancy was present in a majority of patients. The mean duration of treatment was 12.4 (± 2.9) days. Mean time to defervescence was 3.7 days. The most common empiric regimen was cefepime + vancomycin (64.9%) with 78.9% of patients receiving ceftriaxone as definitive therapy. All VGS isolates were susceptible to at least one drug in their empiric regimen. Clinical outcomes are depicted in TABLE 2. There was 1 recurrence of VGS bacteremia and three deaths in the cohort. In TABLE 3, results are stratified by treatment duration.

**Figure:**
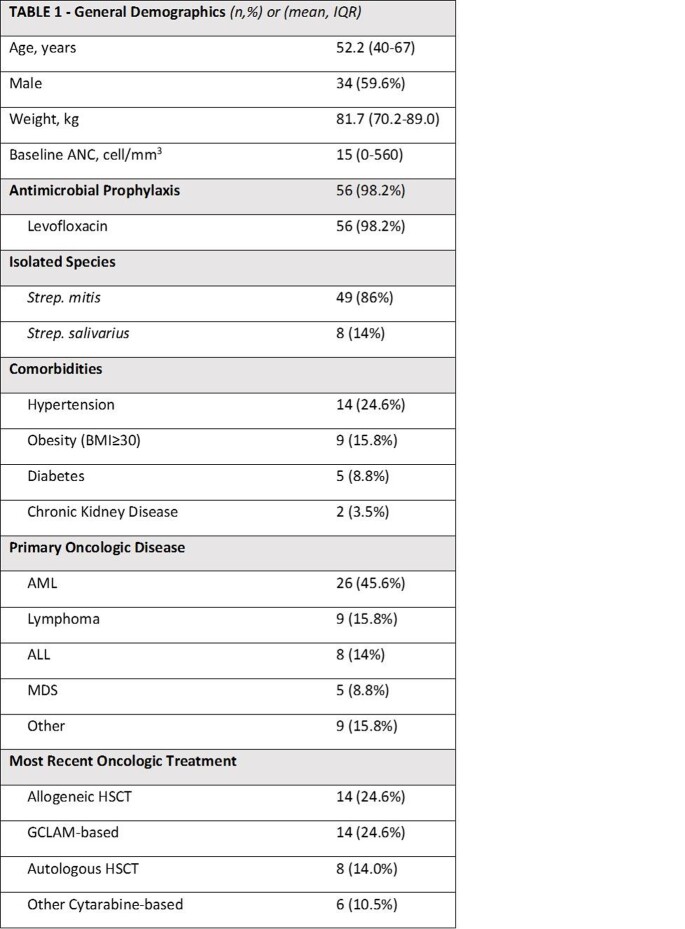

**Figure:**
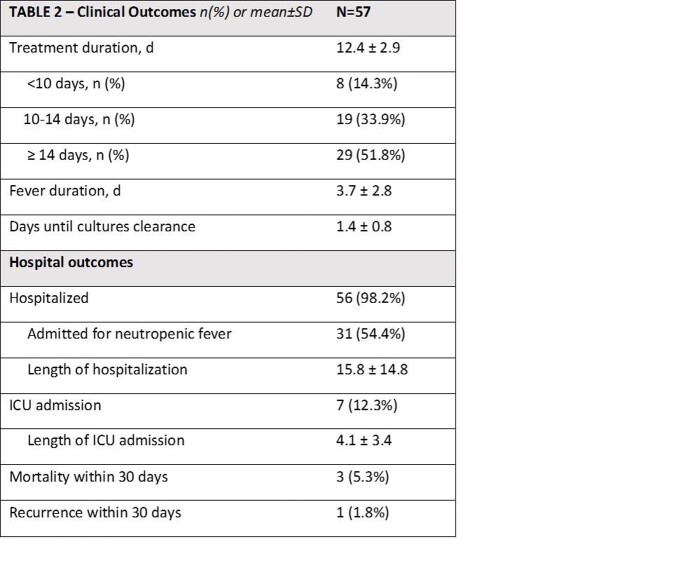

**Figure:**
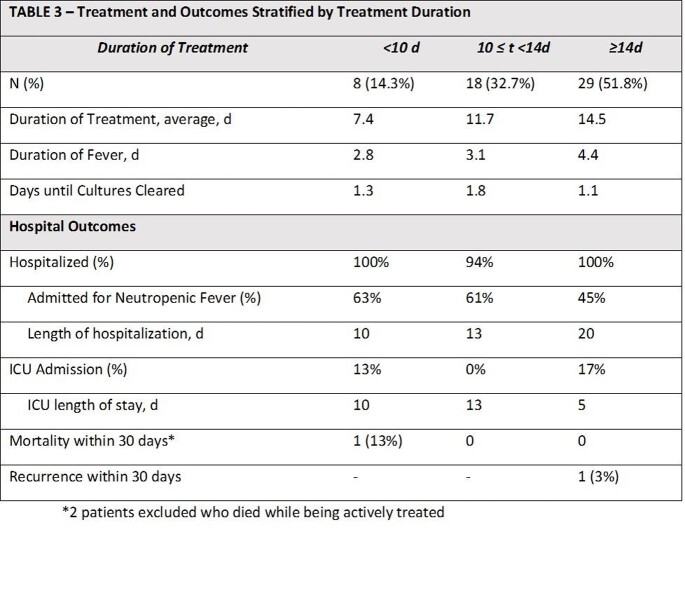

**Conclusion:**

There was significant heterogeneity in the duration of treatment of VGS bacteremia among neutropenic cancer patients utilizing levofloxacin prophylaxis, with > 50% treated in excess of 14 days. Fever persisted for several days despite active antimicrobials consistent with prior reports. The low rate of recurrence and death at any treatment duration suggests that a shorter duration of less than 14 days for VGS bacteremia may be feasible. A prospective evaluation is needed to confirm these findings.

**Disclosures:**

**All Authors**: No reported disclosures.

